# Mercury Toxicity: A Family Case Report

**DOI:** 10.5152/tjh.2011.16

**Published:** 2012-03-05

**Authors:** Rahşan Yıldırım, Fuat Erdem, Mehmet Gündoğdu, Yusuf Bilen, Ebru Koca, Yalçın Yıllıkoğlu, Yaşar Nuri Şahin

**Affiliations:** 1 Atatürk University, School of Medicine, Department of Internal Medicine, Division of Hematology, Erzurum, Turkey; 2 Erzurum Region Education and Research Hospital, Department of Hematology, Erzurum, Turkey; 3 Atatürk University, School of Medicine, Department of Neurology, Erzurum, Turkey; 4 Atatürk University, School of Medicine, Department of Biochemistry, Erzurum, Turkey

**Keywords:** Mercury intoxication, Family

## Abstract

**Background:** Environmental pollution exposes humans to toxic substances. Herein we present 5 family members aged20-54 years that were poisoned by liquid mercury.

**Case Reports:** Case 1 presented to our clinic with cough, fever, and night sweats. The patient had neutropenia, anemia,and pneumonia, rapidly developed acute respiratory distress syndrome (ARDS), and died on day 4 of hospitalization.Her WBC count was 0.4 × 10³ mm-3 (normal range: 4.3-10.3 × 103 mm-3) and Hb was 10.8 g dL–1 (normal range: 11.5-16.0 g dL–1). Case 2 presented with bicytopenia; the leukocyte count was 1.3 × 103 mm-3 (normal range: 4.3-10.3 × 103mm-3) and the PLT count was 88 × 103 mm-3 (normal range: 150-400 × 103 mm-3). Cases 2 and 3 had toxic peripheralneuropathy. The PLT count in case 3 was 123 × 103 mm-3 (normal range: 150-400 × 103 mm-3). Cases 4 and 5 presentedwith fatigue and headache; these 2 patients did not have positive findings, apart from high levels of mercury in theblood. We have written informed consent.

**Conclusion:** We think that heavy metal exposure—although rare—should be considered in patients that present withnumerous symptoms involving multiple systems, including the cardiovascular, respiratory, and neurological systems.The present report is unique in that in describes mercury poisoning in 5 members of the same family.

## Abstract (tr)

**Giriş:** Çevresel kirlilik insanları zehirli maddelere maruz bırakır. Biz, civa ile zehirlenen, yaşları 20-54 arasında olan aynı aileden 5 olguyu sunduk.

**Olgular:** Olgu 1; kliniğimize öksürük, ateş ve gece terlemesi ile başvurdu. Nötropeni, anemi ve pnömonisi olan hastada hızlı bir şekilde akut respiratuar distress sendromu (ARDS) gelişti ve hastaneye yatışının 4. gününde öldü. Hastanın WBC sayısı 0.4 × 10^3^ mm^-3^ (normal sınır: 4.3-10.3 × 10^3^ mm^–3^) ve Hb değeri 10.8 g dL–1 (normal sınır: 11.5-16.0 g dL–1) idi. Olgu 2; bisitopeni ile başvurdu, lökosit sayısı 1.3 × 10^3^ mm^-3^ (normal sınır: 4.3-10.3 × 10^3^ mm^-3^) ve trombosit sayısı 88 × 10^3^ mm^–3^ (normal sınır: 150-400 × 10^3^ mm^–3^) idi. Olgu 2 ve 3’te toksik periferik nöropati tespit edildi. Olgu 3’te trombosit sayısı 123 × 10^3^ mm^-3^ (normal sınır: 150-400 × 10^3^ mm^-3^) idi. Olgu 4 ve 5 halsizlik ve başağrısı ile başvurdu, kandaki yüksek civa seviyeleri dışında bu iki hastada herhangi bir pozitif bulguya rastlanmadı.

**Sonuç:** Biz, kardiyovasküler, solunum ve nörolojik sistem gibi birçok sistemi tutan ve çeşitli semptomlar ile başvuran hastalarda oldukça nadir olsa da ağır metal maruziyetinin değerlendirilmesi gerektiğini düşünüyoruz. Sunulan rapor, aynı ailenin 5 bireyinde civa zehirlenmesi tespit edilmesi sebebi ile tektir.

## INTRODUCTION

Mercury exists in 2 forms—organic and inorganic— both of which are hazardous to human health [1]. Mercury is the only metal that is a liquid at room temperature [2]. Organic liquid mercury is dangerous, especially to children, as it can easily be found in the environment [1]. The components of mercury are lipophilic—they readily penetrate cell membranes and accumulate in several organs, and exhibit toxic activity in numerous systems, including the urinary, central nervous, endocrine, and gastrointestinal systems [3]. Methylmercury, the soluble form of mercury, is neurotoxic [1]. Reports of mercury intoxication vary according to the type of mercury, type of exposure, duration of exposure, and individual sensitivity [4,5]. Herein we present 5 family members aged 20-54 years that were poisoned by liquid mercury and presented with varying symptoms.

## CASE 1

A 54-year-old female presented to our clinic with fever, cough, night sweats, weight loss, and pain in the extremities, all of which began 10 d earlier. Two weeks earlier, the youngest son of the family came home with an unknown putty-like substance he obtained from his school’s science lab, which was subsequently determined to be mercury. The mercury spilled on the floor of the house and 2 weeks later the mother cleaned it up and poured it down the sink; as such, the family was exposed via inhalation and/or dermal contact during a 2-week period.

The results of physical examination in case 1 were as follows: temperature: 39.2 °C (axillary); blood pressure: 130/80 mmHg (brachial); pulse rate: 120 bpm (radial and rhythmic); respiratory rate: 25 min–^1^; O2 saturation at room temperature based on pulse oximetry: 88%. Examination of the head, neck, and abdomen, and cardiovascular and neurological systems was unremarkable. Pulmonary auscultation showed bilateral inspiratory rales in the lower zones. Whole blood analysis results were as follows, with normal ranges in parentheses: leukocyte count: 0.4 x 10^6^ L–^1^ (4.3-10.3 x 10^6^ L–^1^); Hb: 10.8 g dL–^1^ (11.5-16.0 g dL–^1^); thrombocyte count: 173 x 10^9^ L–^1^ (150-400 x10^9^ L–^1^); MCV: 79 fL (80-100 fL); erythrocyte sedimentaThe results of physical examination in case 1 were as follows: temperature: 39.2 °C (axillary); blood pressure: 130/80 mmHg (brachial); pulse rate: 120 bpm (radial and rhythmic); respiratory rate: 25 min–^1^; O2 saturation at room temperature based on pulse oximetry: 88%. Examination of the head, neck, and abdomen, and cardiovascular and neurological systems was unremarkable. Pulmonary auscultation showed bilateral inspiratory rales in the lower zones. Whole blood analysis results were as follows, with normal ranges in parentheses: leukocyte count: 0.4 x 10^6^ L–^1^ (4.3-10.3 x 10^6^ L–^1^); Hb: 10.8 g dL–1 (11.5-16.0 g dL–^1^); thrombocyte count: 173 x 10^9^ L–^1^ (150-400 x10^9^ L–^1^); MCV: 79 fL (80-100 fL); erythrocyte sedimentation rate: 120 mm h–^1^ (0-20 mm h–^1^). Serum biochemistry parameters were as follows, with normal ranges in parentheses: creatinine kinase: 574 U L–^1^ (<145 U L–^1^); aspartate aminotransferase: 30 U L–^1^ (<31 U L–^1^); alanine aminotransferase: 37 U L–^1^ (<34 U L–^1^); albumin: 1.9 g dLAutopsy was not performed because the patient’s firstdegree

relatives did not consent. The (3.5-5.2 g dL–^1^); BUN: 44 mg dL–^1^ (6-22 mg dL–^1^); creatinine: 2.0 mg dL–^1^ (0.6-1.09 mg dL–^1^); Na: 132 mmol L–^1^ (135-145 mmol L–^1^); potassium: 2.5 mmol L–^1^ (normal range: 3.5-5.5 mmol L–^1^); lactate dehydrogenase: 349 IU L–^1^ (150-450 IU L–^1^); C-reactive protein: 19.9 mg L–^1^ (0-0.5 mg L–^1^). Other parameters were within the normal range. Posteroanterior (PA) chest radiography showed a bilateral non-homogenous density increase in the basals. 

**The blood mercury level was**

518 μg dL–^1^ and the urea mercury level was 180 μg dL–^1^ (normal range: 0-10 μg dL–^1^, in accord with the hospital laboratory reference). The blood mercury level was measured via the hydride method using an atomic absorption machine. In addition to chelation treatment with British anti-Lewisite (BAL) (2.5 mg kg–^1^) and N-acetyl cysteine (NAC), antibiotherapy and granulocyte colony-stimulating factors (GCSF) were administered.

The patient’s general condition deteriorated on day 2 of hospitalization and her respiratory rate increased to 35 min–^1^. Arterial blood gas analysis showed severe hypoxemia. Her P O2/FiO2 was <200 mmHg. Follow-up PA radiography showed a progressive increase in bilateral density, as compared to the previous radiograph. Considering acute respiratory distress syndrome (ARDS), the patient was connected to a mechanical ventilator. The patient developed cardiac arrest and died on day 2 of mechanical ventilation.

Autopsy was not performed because the patient’s firstdegree relatives did not consent. The next 4 cases [2,3,4,5] presented with varying symptoms 1 week after case 1. All 5 cases were relatives living in the same house. Case 1 was the mother, case 2 was a son of case 1, case 3 was a daughter- in-law, and cases 4 and 5 were daughters of case 1.

## CASE 2

A 29-year-old male presented to our clinic with headache, gingival pain, and numbness in the arms and legs.

There were no relevant findings noted during examination of all systems. Whole blood analysis results were as follows, with normal ranges in parentheses: leukocyte count: 1.3 L–^1^ (4.3-10.3 x 10^6^ L–^1^); Hb 13.6 dL–^1^ (11.5-16.0 g dL–^1^); PLT count: 88 x 10^9^ L–^1^ (150-400 x 10^9^ L–^1^); MCV: 85 fL (80-100 fL). The erythrocyte sedimentation rate was 45 mm h–^1^ (normal range: 0-20 mm h–^1^). Serum biochemistry parameters were as follows, with normal ranges in parentheses: aspartate aminotransferase 49 L–^1^ (<31 U L–^1^); alanine aminotransferase 103 L–^1^ (<34 U L–^1^). Other parameters were within the normal range. Blood and urea mercury levels were high—54.4 μg dL–^1^ (normal range: 0-10 μg dL–^1^) and 140 μg dL–^1^, respectively.

Electromyography (EMG ) was performed due to severe numbness in the extremities, and showed rare fibrillation potentials and dense fasciculation muscle potentials in the distal muscles. These findings were considered indicative of toxic neuropathy. In addition to chelation treatment, GCSF, gabapentin, and vitamin B complex were also administered. On day 12 of hospitalization the patient’s leukocyte and PLT counts, and liver function were normal and a marked decrease in extremity numbness was noted. The patient was prescribed gabapentin and vitamin B complex, and then discharged.

## CASE 3

A 22-year-old female presented to our clinic with headache, gingival pain, and numbness in the arms and legs that began 1 week earlier. There were no relevant findings noted during examination of all systems. Whole blood analysis results were as follows, with normal ranges in parentheses: leukocyte count: 5 L–^1^ (4.3-10.3 x 10^6^ L–^1^); Hb: 10.6 dL–^1^ (11.5-16.0 g dL–^1^); PLT count: 123 x 10^9^ L–^1^ (150-400 x 10^9^ L–^1^); MCV: 83 fL (80-100 fL). Biochemical liver and kidney function tests were normal. The blood mercury level was 12.99 μg dL–^1^ (normal range: 0-10 μg dL–^1^). EMG showed rare fibrillation potentials and dense fasciculation muscle potentials in the distal muscles. These findings were considered indicative of toxic neuropathy. Chelation treatment, gabapentin, and vitamin B complex were administered. On day 10 of hospitalization the patient’s Hb and PLT levels were normal. The patient’s symptoms subsided and she was discharged with a prescription for gabapentin and vitamin B complex.

## CASE 4 AND 5

A 20-year-old female (case 4) and 23-year-old female (case 5) presented to our clinic with fatigue and headache. There were no relevant findings noted during examination of all systems. Whole blood analysis and biochemical tests were normal. Blood mercury levels were high—51 μg dL–^1^ and 43 μg dL–^1^ (normal range: 0-10 μg dL–^1^), respectively. EMG was normal. Chelation treatment was administered. Both patients were discharged without any symptoms of disease.

## DISCUSSION

Mercury is a liquid element that is easily vaporized and inhaled at room temperature [6]. Acute inhalation of mercury vapors can lead to pneumonia, ARDS, progressive pulmonary fibrosis, and death. In addition, elemental (metallic) mercury can easily enter systemic circulation directly via the skin or inhalation of mercury vapors via the alveoli [7]. Universally, chelating agents, including dimercaptosuccinic acid (DMSA), dimercaprol (BAL), and 2,3-dimercaptopropane-1-sulphonate (DMPS), are used to treat mercury intoxication [1].

The presented family case included 5 members with elemental mercury intoxication due to inhalation of vapors and/or dermal contact. Case 1 suffered the most extensive exposure, and as such her clinical presentation was the most severe and she died. Clinical presentation in the other 4 cases was less severe, as they were exposed to a lesser degree. The literature contains reports of mercury poisonings that occurred in hospitals, homes, and schools due to broken thermometers, sphygmomanometers, and barometers containing mercury [8,9]. Mercury poisoning was diagnosed in 3 students that were examined due to hypertension; 2 of the students had rash, peripheral neuropathy, mild and moderate proteinuria, and central nervous system involvement [10]. Case 1 in the present study presented with febrile neutropenia and pneumonia, and she rapidly progressed to ARDS, and then died. Cases 2 and 3 had toxic peripheral neuropathy, which was confirmed via EMG . EMG findings in case 4 were normal and EMG could not be performed in case 5 due to technical reasons. GCSF was used to treat cases 1 and 2 due to neutropenia. Neutropenia in case 2 resolved with chelation treatment and GCSF within 10 d, but in case 1 (with more severe exposure) the disease progressed and she died. Anemia and thrombocytopenia were noted in case 3. With normal levels of leukocyte, ferritin, and vitamin B12, laboratory findings returned to normal values with chelation treatment. Clinical symptoms in all 5 cases were confirmed to be due to mercury exposure based on urea and/or whole blood analysis.

Mercury quickly evaporates and pollutes the air we breathe. Glass mercury thermometers can break in the mouth, causing the inhalation and/or ingestion of mercury. Proper disposal of fluorescent light bulbs, which also contain mercury, and mercury thermometers is essential; they shouldn’t simply be put out with the trash—in fact most cities and towns have ordinances prohibiting such disposal. The proper authorities must handle any spill using appropriate mercury decontamination kits and procedures.

The presented study reported the varying clinical presentation of 5 family members exposed to liquid mercury via dermal contact and/or inhalation. Although a rare occurrence, we think that heavy metal exposure should be considered in patients that present with numerous symptoms involving multiple systems, including the skin, and cardiovascular, respiratory, and neurological systems. This family case report is unique in that it included 5 members of the same family.

## CONFLICT OF INTEREST STATEMENT

The authors of this paper have no conflicts of interest, including specific financial interests, relationships, and/ or affiliations relevant to the subject matter or materials included.
